# Clinical analysis of the MyoSure hysteroscopic tissue removal system of endometrial polyps in women with an intact hymen

**DOI:** 10.1186/s12905-021-01362-w

**Published:** 2021-05-22

**Authors:** Jiahui Yong, Xiaohui Guo, Hua Lan, Jing Yuan, Da Zeng, Xiangyang Zeng, Shuijing Yi, Songshu Xiao

**Affiliations:** Department of Gynecology and Obstetrics, Third Xiangya Hospital, Central South University, Changsha, 410013 Hunan China

**Keywords:** Hysteroscopic tissue removal system, MyoSure, Endometrial polyps, Cervical polyps, Women with an intact hymen

## Abstract

**Background:**

To investigate the clinical efficacy of the MyoSure hysteroscopic tissue removal system in the treatment of endometrial and cervical polyps in women with an intact hymen.

**Methods:**

Retrospective analysis was performed on the clinical data of 32 patients treated with the MyoSure hysteroscopic tissue removal system for endometrial and cervical polyps.

**Results:**

All the patients successfully completed the procedure. No intraoperative complications, such as cervical trauma, uterine perforation or TURP syndrome, were reported. The surgical time ranged from 5 to 35 min, with an average time of 19.3 min, and the intraoperative blood loss ranged from 2 to 50 ml with an average blood loss of 10.8 ml. After surgery, all patients were shown to have intact hymens. No residual polyp tissues were observed under the microscope, and abnormal uterine bleeding was relieved.

**Conclusions:**

The MyoSure hysteroscopic tissue removal system can be a safe and effective treatment for endometrial and cervical polyps in women with an intact hymen.

## Introduction

Endometrial polyps are benign intrauterine lesions caused by local overgrowth of the endometrium which account for 21–39% of abnormal uterine bleeding. Between 70 and 90% of patients present with intermenstrual bleeding, increased menstrual volume, prolonged menstrual periods, or irregular vaginal bleeding [1]. Hysteroscopic resection combined with pathological examination seems to be the gold standard for the diagnosis of endometrial polyps. However, for women with intact hymens, it is extremely difficult to operate through the vagina and requires a surgeon with high technical ability.

In traditional hysteroscopy, we often use speculum for a better visibility and facilitate the procedure. But for women with an intact hymen, the use of the instrument may damage the hymen, of which the integrity is an important representation of virginity in our country. For these reasons, patients may refuse to take the surgery, which may result in a delayed diagnosis or even improper management.

The MyoSure hysteroscopic tissue removal system is a rotary tubular intrauterine lesion excision system that has shown high efficiency and safety in the treatment of benign intrauterine lesions, including submucosal myomas, retained products of conception (RPOC) and endometrial polyps [2–5]. In this retrospective study, we evaluated the results of patients with an intact hymen who underwent MyoSure for the resection of intrauterine and cervical polyps under the condition of ensuring the integrity of the hymen, and found its unique advantages.

## Materials and methods

### Study site

The research was conducted at the Department of Gynecology and Obstetrics, The Third Xiangya Hospital, Central South University. The study was approved by the Ethics Committee of the Third Xiangya Hospital.

### Patient

From December 2014 to May 2020, a total of 32 patients were included in the study. The inclusion criteria consisted of patients who (1) manifested with abnormal uterine bleeding, treated with normal hormone therapy for 3–6 months but was ineffective or effective during the drug treatment but after which the symptoms reappeared in a short period of time; (2) had no history of sexual behavior and were confirmed by gynecological examination to have an intact hymen; (3) had intrauterine lesions or intracervical lesions identified by preoperative ultrasound; (4) had no accompanying blood system diseases, liver or kidney dysfunction; and (5) had no contraindications for hysteroscopic surgery. The exclusion criteria were as follows: (1) patients with acute systemic or pelvic inflammatory diseases and (2) patients with malignant tumors of the reproductive tract.

The clinical information on the 32 patients included age, clinical manifestations, preoperative hemoglobin and preoperative evaluation of the lesions on ultrasound (Table 1).

**Table 1:** Baseline characteristics of the patients

Variables	Entire cohort (n = 32)
Age (years)	24.88 ± 6.61
Clinical manifestation	
Intermenstrual bleeding	19
Increased menstrual volume/prolonged menstrual period	7
Irregular vaginal bleeding	6
Preoperative hemoglobin (g/l)	111.63 (60.00,144.00)
Mild anemia	7
Moderate anemia	6
Abnormal intrauterine echo size (cm^2^)	0.4 × 0.5–1.4 × 2.1

### Surgical procedure

Hysteroscopic resection of intrauterine and cervical lesions was performed by experienced gynecologists. After general anesthesia, the vulva was cleansed with povidone iodine, which was introduced into the vagina through a bladder catheter. Distension of the uterine cavity was performed using 0.9% normal saline. Intrauterine pressure was automatically controlled by an electronic irrigation and suction device, which was set at 100–120 mmHg. The procedure was initiated by insertion of the 4.5-mm hysteroscope into the vagina. The vaginal wall and cervix were visualized to determine whether there were any lesions. Then, the cervical canal and uterine cavity were gradually checked for lesions. Then, we performed all procedures using the MyoSure (Lite type, 2.5 mm inner blade, 6.25 mm hysteroscope outer diameter) hysteroscopic tissue removal system (Hologic, Marlborough, MA). After the surgery, 3 ml self-crosslinking sodium hyaluronate gel (Bioregen, Changzhou, China) was injected into the uterine cavity to prevent intrauterine adhesion. The patient's vital signs, vaginal bleeding, abdominal pain and other conditions were closely monitored. Patients who had vaginal bleeding for more than 7 days before the operation were given 0.5 g cephalosporins bid × 3 days after the operation to prevent infection. All excised tissue was sent for pathological examination. Once the pathological examination confirmed the endometrial polyps, patients were given hormone therapy for regulation of menstrual cycle (second half of menstrual cycle with 200 mg oral progesterone Qd or second half of menstrual cycle with 10 mg oral dydrogesterone bid for a total of three menstrual cycles).

### Clinical evaluation

Clinical evaluation data included perioperative parameters (operative time, intraoperative blood loss and perioperative complications), hysteroscopic evaluation accompanied by preoperative findings under the ultrasound of the characteristics of the polyps (type, location and size) and examination of the integrity of the hymen after the surgery. Among which, the operative time was defined as from the beginning to the end of the operation, and the intraoperative blood loss was measured by observing the results of blood-gas analysis and comparing the value of preoperative and postoperative hemoglobin.

### Follow-up

After the first and third menstruation, patients were examined by pelvic ultrasound to determine if there was recurrence of polyps. Abnormal uterine bleeding and menstrual recovery were also assessed during follow-up.

### Statistical analysis

Data were presented as mean ± SD or median (minimum, maximum) of cohort. Data were analyzed using Excel 2010 (Microsoft Corp., Redmond, WA, USA).

## Results

**Fig. 1:**
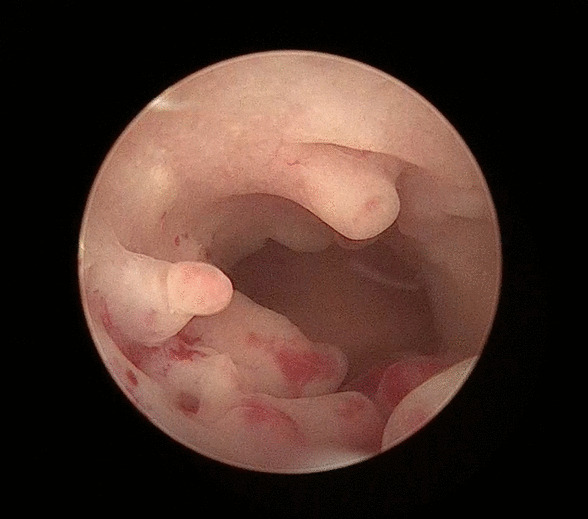
Multiple polyps in the intrauterine cavity

**Fig. 2:**
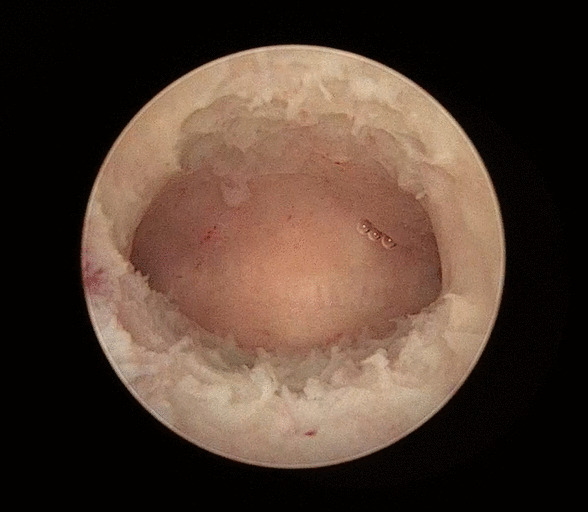
No residue of polyps in the intrauterine cavity

All the endometrial polyps or cervical polyps in 32 patients were successfully resected using MyoSure. No perioperative complications, such as cervical trauma, uterine perforation or TURP syndrome, were reported. The operative time ranged from 5 to 35 min, with an average time of 19.3 min, and intraoperative blood loss ranged from 2 to 50 ml with an average blood loss of 10.8 ml. Endometrial polyps were found in a total of 31 patients, of which the maximum diameter of the lesion was (1.52 ± 0.68) cm, among which 8 cases were single endometrial polyps, and another 23 cases were multiple endometrial polyps (Fig. 1). Six patients had cervical polyps, and the maximum lesion diameter was (2.33 ± 1.51) cm. After surgery, all patients were shown to have intact hymens. No residual polyp tissues were identified microscopically, and abnormal uterine bleeding was relieved (Fig. 2).

## Discussion

Endometrial polyps are one of the most common intrauterine lesions in women of childbearing age. Most patients are treated for abnormal uterine bleeding, irregular vaginal bleeding, infertility or other causes. Treatment for endometrial polyps includes conservative management, medical management and surgical removal. Expectant management is suitable for functional polyps, which can shed with menstrual blood. Medical management includes the application of hormonal therapies, such as using oral progesterone during the second half of the menstrual cycle or taking oral contraceptives, but at the same time, side effects related to the long-term use of hormonal drugs, such as endocrine disorders, should not be ignored. For cervical polyps, using merely medical therapy is unlikely to achieve a radical cure. Hysteroscopy and electrosurgical removal of polys are recommended for patients with large polyps or those who, after ineffective treatment with hormonal drugs or who have achieved effective treatment, experience recurrence within a short period of time. However, for women without sexual activity, performing hysteroscopic electropolypectomy is very difficult. Only a few cases have discussed the protection of hymen integrity during surgery [6]. Victoria reported a case of hysteroscopic resection of endometrial polyps in a twelve-year-old girl. Polyp tissue was completely removed after the second surgery, and finally, the symptom of abnormal uterine bleeding was relieved [7]. Cheong performed hysteroscopic resection of a cervical polyp that protruded from the vagina in a virgin [8]. We started hysteroscopic resection of intrauterine lesions in women without sexual activity in 2003 and have achieved much experience [9].

MyoSure has achieved great efficacy since its application in 2014 for benign intrauterine lesions in women with intact hymens in our hospital. MyoSure has unique advantages. The external diameter of the electroresectoscope is 9–10 mm, and the cervix is softened before the procedure. Additionally, the operation requires high technical requirements of the surgeon. However, the external diameter of the MyoSure instrument is 6.25 mm, and there is no need to soften the cervix before surgery. During surgery, lesions were resected with a high-speed rotating blade inside the sidewall of the instrument, and only fine adjustment of the lens body was needed to complete the operation, which greatly reduced the probability of damaging the hymen. At the same time, the suction device is attached after the operating handle of MyoSure. After polypectomy, polyp fragments can be removed and stored in the specimen storage tank at the same time, ensuring clarity of the surgical field and reducing the number of times that the mirror body enters and leaves the uterine cavity, preserving the intact hymen. In addition, MyoSure's cutting window is located on the sidewall of the instrument, which limits the surgical field. Additionally, it uses mechanical energy to avoid electric and heat damage, which protects the membrane of the normal parts around the lesion and reduces the occurrence of long-term postoperative complications, such as intrauterine adhesion. Additionally, the cutting depth of the electrosurgical ring is difficult to control, which increases the risk of cervical trauma and uterine perforation. However, the cutting window of MyoSure is parallel to the base of polyps, which avoids cervical damage. In our study, all endometrial polyps or cervical polyps in the 32 patients were successfully removed. Intraoperative blood loss and operative duration were minimized. No intraoperative complications, such as cervical injury, uterine perforation or TURP syndrome, occurred. Hymen integrity was preserved, and symptoms of abnormal uterine bleeding were relieved. MyoSure resection of endometrial polyps and cervical polyps is safe and efficient, allowing preservation of the intact hymen.

Diagnosis and treatment of intrauterine lesions in women without sexual behavior has particularities. However, if the indications for surgery are strictly grasped and the perioperative option is considered, satisfactory effects can be obtained. First, women with intact hymens are prone to resistance and fear transvaginal uterine operations. Patients should be fully informed of the necessity and risk of surgery before surgery. General anesthesia is recommended during the surgery, and the operation should be performed after a satisfactory anesthesia effect to avoid hymen injury caused by repeated movement by the patient. Second, the complex iodine rinse for disinfection of the vagina can be rejected through a catheter. The distension fluid can also be used for expanding and flushing the vagina to prevent bacteria from retrograding into the uterine cavity and even causing pelvic inflammation. In addition, although there is no need to soften the cervix before surgery, movement of the instrument should be controlled. Caution should be taken regarding both the entry and exit of the instrument to avoid complications and protect the integrity of the hymen.

Study strengths included the discussion of the advantages of MyoSure hysteroscopic tissue removal system as a new attempt in the treatment of endometrial and cervical polyps in women with an intact hymen. Primary limitations of this study included the fact that data were retrospectively collected and the sample size was relatively small. Also, this is a single-center study, which limits the option to generalize its conclusions. Further studies are needed to confirm the advantages of MyoSure tissue removal system in these particular types of cases, and to explore more options for treatment of intrauterine lesions in women with an intact hymen.

In conclusion, MyoSure tissue removal system can be a safe and efficient option for the treatment of endometrial polyps in women with an intact hymen. Under the condition of strictly grasping the surgical indications and matters needing attention, MyoSure can effectively remove the intrauterine lesions, and preserve the integrity of the hymen.

## Data Availability

The datasets used and analysed during the current study are available from the corresponding author.
